# Bovine Lhx8, a Germ Cell-Specific Nuclear Factor, Interacts with Figla

**DOI:** 10.1371/journal.pone.0164671

**Published:** 2016-10-07

**Authors:** Liyuan Fu, Mingxiang Zhang, Kristen Mastrantoni, Mark Perfetto, Shuo Wei, Jianbo Yao

**Affiliations:** 1 Division of Animal and Nutritional Sciences, West Virginia University, Morgantown, West Virginia, United States of America; 2 Department of Biology, West Virginia University, Morgantown, West Virginia, United States of America; University of Texas at Austin Dell Medical School, UNITED STATES

## Abstract

LIM homeobox 8 (Lhx8) is a germ cell-specific transcription factor essential for the development of oocytes during early oogenesis. In mice, Lhx8 deficiency causes postnatal oocyte loss and affects the expression of many oocyte-specific genes. The aims of this study were to characterize the bovine *Lhx8* gene, determine its mRNA expression during oocyte development and early embryogenesis, and evaluate its interactions with other oocyte-specific transcription factors. The bovine *Lhx8* gene encodes a protein of 377 amino acids. A splice variant of *Lhx8* (*Lhx8_v1*) was also identified. The predicted bovine Lhx8 protein contains two LIM domains and one homeobox domain. However, one of the LIM domains in Lhx8_v1 is incomplete due to deletion of 83 amino acids near the N terminus. Both *Lhx8* and *Lhx8_v1* transcripts were only detected in the gonads but none of the somatic tissues examined. The expression of *Lhx8* and *Lhx8_v1* appears to be restricted to oocytes as none of the transcripts was detectable in granulosa or theca cells. The maternal *Lhx8* transcript is abundant in GV and MII stage oocytes as well as in early embryos but disappear by morula stage. A nuclear localization signal that is required for the import of Lhx8 into nucleus was identified, and Lhx8 is predominantly localized in the nucleus when ectopically expressed in mammalian cells. Finally, a novel interaction between Lhx8 and Figla, another transcription factor essential for oogenesis, was detected. The results provide new information for studying the mechanisms of action for Lhx8 in oocyte development and early embryogenesis.

## Introduction

A well-ordered series of events coordinating multiple molecular pathways regulate oogenesis and embryogenesis. Transcription factors play important roles during these developmental processes. They control the expression of target genes due to their varying DNA-binding specificities [1], and the specificity of target DNA-binding is achieved through selective protein interactions. Among different categories of transcription factors, germ cell-specific transcription factors are essential for follicle formation and subsequent development [2]. Examples of such factors include LIM homeobox 8 (Lhx8) [3], factor in germline alpha (Figla) [4], spermatogenesis and oogenesis specific basic helix-loop-helix 1 (Sohlh1) [5], and newborn ovary homeobox (Nobox) [6]. Deficiencies of these genes in mice lead to failure of primordial follicle formation or disruption of early follicular development.

Lhx8 is a germ cell-specific transcription factor highly expressed in mammalian ovaries [7, 8]. In mice, Lhx8-null ovaries fail to maintain primordial follicles due to loss of oocytes within 7 days. Many oocyte-specific genes, such as *Gdf9*, *Pou5f1*, and *Nobox*, are misexpressed in Lhx8-null ovaries [3]. Lhx8 is a LIM homeobox protein containing two tandem LIM domains and one homeobox domain. The LIM domain is a unique double-zinc finger motif which mediates protein-protein interactions, and can interact with other LIM domain-containing proteins [9–14]. Homomeric or heteromeric complexes are formed when LIM domain interact with other regulatory proteins [15, 16]. LIM homeobox proteins may also interact with ring finger proteins through the LIM domains, causing repression of gene expression [17]. These studies suggest that LIM containing proteins exert their functions through interactions with other proteins. As a germ cell-specific transcription factor, Lhx8 may also regulate target genes by context-dependent protein interactions.

Figla is a basic helix-loop-helix (bHLH) transcription factor that is essential for folliculogenesis and activation of oocyte-associated genes during normal oogenesis. In mice, ablation of *Figla* gene causes female sterility due to failure in the formation of primordial follicles [4]. Sohlh1 is also a germ cell-specific bHLH transcription factor. Female mice lacking *Sohlh1* have normal germ cell migration and embryonic gonadogenesis, but form imperfect primordial follicles that do not progress to primary follicles [5]. *Sohlh1*-null ovaries contain significantly less *Figla* mRNA and transcripts for the Figla target genes, *Zp1* and *Zp2* [5]. Despite their important roles in folliculogenesis, little is known about the mechanisms of action for Figla and Sohlh1, and it is not clear if they interact with other nuclear proteins to exert their functions.

The major objectives of this study were to clone and analyze the bovine *Lhx8* cDNA and protein sequences, determine the temporal expression of *Lhx8* transcripts during oocyte maturation and early embryonic development, and identify protein partners that directly interact with Lhx8 protein in vitro and in vivo. We show that expression of bovine *Lhx8* is temporally regulated during oogenesis and early embryonic development, and Lhx8 interacts with Figla. To our knowledge, this is the first time that a direct protein-protein interaction between two germ cell-specific transcription factors essential for oocyte and follicular development is demonstrated.

## Materials and Methods

### Tissue sample collection

Bovine tissue samples were collected from slaughtered cattle at a local slaughterhouse (no animals were killed specifically for this study). These samples include spleen, stomach, brain, muscle, kidney, liver, heart, intestine, adult ovary, adult testis, fetal testis and fetal ovary. Fetal ovaries from different gestation stages were collected and the ages of fetuses were estimated based on crown-rump length [18]. Granulosa and theca cells were dissected from antral follicles using an established method [19]. Briefly, antral follicles were isolated and frozen in liquid nitrogen. A 3-quarter cut around the circumference of the follicle was made and the outer-theca layer was removed by peeling with forceps, leaving the granulose layer that is still adhered to the frozen follicular fluid core. Samples after collection were frozen in liquid nitrogen and subsequently stored in -80°C until use.

### RNA isolation, cDNA synthesis, and RT-PCR analysis

Total RNA from different tissues was isolated using Trizol (Invitrogen, Carlsbad, CA) according to the manufacturer’s instructions followed by DNase treatment. Oocyte and embryo samples, including GV- and MII-stage oocytes and 2-cell, 4-cell, 8-cell, 16-cell, and morula- and blastocyst-stage embryos (20 oocytes/embryos), were purchased from Bomed, Inc. Total RNA from oocytes and embryos was isolated and simultaneously subjected to DNase treatment using the RNAqueous Micro Scale RNA isolation kit (Ambion, Austin, TX). The DNase-treated RNA was converted to cDNA using oligo (dT)_18_ primer and SuperScript III reverse transcriptase (Invitrogen).

For cloning of bovine *Lhx8* cDNA, PCR was performed to amplify different regions of the cDNA using primers (Table 1) designed based on a predicted bovine *Lhx8* sequence and a partial bovine *Lhx8* 5’ transcript. The amplified cDNA fragments were cloned into pGEM-T-easy vector (Promega, Madison, WI) and sequenced.

**Table 1:** Primers used in this study.

Primer name	Primer sequence
*Cloning Lhx8 into AD/BD vector*	
bLhx8inADBDF	GGCCGAATTCATGTTTGTGTGTAAACTAGAAG
bLhx8inADBDR	GGCCGGATCCTTAGGTATGACTTATTGGCAG
bFiglainADF	GGCCCATATGGACGCCGCGCCCGAGCTCC
bFiglainADR	GGCCGAATTCCTATCCACTGCCACCATCTGGC
bSohlh1inADF	GGCCCATATGGCGTCCCGGGCTCCTGAG
bSohlh1inADR	GGCCGAATTCTCAGCAGGCGAAGAAGTCGGG
*Cloning Lhx8 into EGFP vector*	
bLhx8inEGFPF	GGCCGAATTCATGTTTGTGTGTAAACTAGAAGA
bLhx8inEGFPR	GGCCCTCGAGGGTATGACTTATTGGCAGTTG
*Two step PCR mutagenesis to delete Monopartite NLS*	
MutmonobLhx8F1	GGCCGAATTCATGTTTGTGTGTAAACTAGAAGA
MutmonobLhx8R1	ATGATTAACATCTTGCTCTGTAAGG
MutmonobLhx8F2	CCTTACAGAGCAAGATGTTAATCAT ACAGCAGATCAGCTCCAGGTTATGC
MutmonobLhx8R2	GGCCCTCGAG GGTATGACTTATTGGCAGTTG
*Two step PCR mutagenesis to delete Bipartite NLS*	
MutbibLhx8F1	GGCCGAATTCATGTTTGTGTGTAAACTAGAAGA
MutbibLhx8R1	GCTCAAGCCTGTCCTTTCTGCCAG
MutbibLhx8F2	CTGGCAGAAAGGACAGGCTTGAGCCATGTCAGTCCTAATCACTCC
MutbibLhx8R2	GGCCCTCGAGGGTATGACTTATTGGCAGTTG
*Cloning LIM domains and homeobox domain*	
bLhx8LIMF	GGCCGAATTCATGTTTGTGTGTAAACTAGAAG
bLhx8LIMR	GGCCGGATCCGTAAGGAGGGCGCCTTCAAC
bLhx8HomeoF	GGCCGAATTCATGGGTAATGGAATTAGTGTTGAAGG
bLhx8HomeoR	GGCCGGATCCTTAGGTATGACTTATTGGCAG
*Cloning expression constructs for Co-IP experiments*	
CoIPbLhx8F	GGCCGGATCCGCCGCCACCATGTTTGTGTGTAAACTAGAAG
CoIPbLhx8R	GGCCGAATTCTTA CTTGTCATCGTCGTCCTTGTAGTCGGTATGACTTATTGGCAGTTG
CoIPbFiglaF	GGCCGGATCCGCCGCCACCATGGACGCCGCGCCCGAGCTCC
CoIPbFiglaR	GGCCGAATTCCTAAGCGTAATCTGGAACATCGTATGGGTATCCACTGCCACCATCTGGCCAG
*RT-PCR analysis of Lhx8 and Lhx8_v1 mRNA expression*	
bLxh8-F-v1	CAGTTCCCCTTGAGAAGGTGA
bLxh8-F	GTCTCATGTCGGAGGAGTGC
bLxh8-R-common	CCAGCATGCAGTCATAATGCA

For gene expression analysis, RT-PCR was performed in a 25-μl reaction using gene-specific primers (Table 1) under the following conditions: 5 min denaturation at 94°C followed by 30 cycles of 94°C for 30 sec, 58°C for 45 sec, and 72°C for 30 sec, and a final extension at 72°C for 10 min. Bovine *RPL19* was used as a control.

### Protein localization assay

The coding region of bovine *Lhx8* was PCR-amplified using gene-specific primers containing EcoRI and XhoI sites (Table 1) and cloned in-frame with the EGFP sequence in pcDNA3-EGFP vector (Addgene, Cambridge, MA) to generate the wild-type *Lhx8* expression construct, pcDNA3-EGFP-Lhx8-wt. *Lhx8* mutants were produced by 2-step PCR using primers (Table 1) designed to create mutants with either the monopartite NLS or the bipartite NLS deleted. Mutant EGFP-Lhx8 constructs (pcDNA3-EGFP-Lhx8-ΔM-NLS and pcDNA3-EGFP-Lhx8-ΔB-NLS) were generated by cloning the *Lhx8* mutants in pcDNA3-EGFP vector. All constructs were confirmed by DNA sequencing.

HEK293 cells were seeded on 20-mm Poly-D-lysine coated German coverslips (Neuvitro, E1 Monte, CA) inside 6-well plates at 5 x 10^5^ cells per well. Twenty four hours after plating, cells were transfected with 1 μg of each plasmid using transfection reagent X-tremeGENE 9 (Roche Diagnostics, Indianapolis, IN). Coverslips containing the cells were washed with PBS 24 h after transfection and fixed in methanol for 5 min followed by DAPI staining. Cells were analyzed using a fluorescence microscope (Zeiss M1 microscope).

### Direct yeast two-hybridization

Protein interactions between Lhx8 and Figla or Sohlh1 was assessed by direct yeast two-hybrid analysis using the Matchmaker Two-Hybrid System (Clontech Laboratories, Mountain View, CA). The coding region of bovine Lhx8 was cloned in-frame into pGBKT7 vector (pGBKT7-Lhx8). The plasmid was transformed into Y2HGold strain cells. The coding regions of bovine *Figla* or *Sohlhl* were cloned in-frame into pGADT7 vector, generating AD constructs (pGADT7-Figla and pGADT7-Sohlhl). The AD constructs were transformed into Y187 cells. All transformants were tested for toxicity and auto-activation before mating according to the manufacturer's protocol. The yeast cells expressing BD-Lhx8 were mated with cells expressing either AD-Figla or AD-Sohlhl fusion proteins. After mating at 30°C for 24 h, yeast cells were plated on DDO/X/A double dropout selection medium (lacking tryptophan and leucine and supplemented with X-α-Gal and Aureobasidin A) plates and incubated at 30°C for 3 days. Single blue colonies (>2mm) were selected and streaked onto fresh QDO/X/A quadruple dropout plates (lacking adenine, histidine, tryptophan and leucine and supplemented with X-α-Gal and Aureobasidin A).

### Co-immunoprecipitation

To confirm the direct interaction between bovine Lhx8 and Figla, as observed by yeast two-hybridization, co-immunoprecipitation (Co-IP) was performed in HEK293 cells co-expressing tagged Lhx8 and Figla proteins. The plasmid expressing Lhx8-FLAG was constructed by cloning the *Lhx8* ORF in frame with a FLAG tag sequence at the 3’-end into pcDNA3.1 (pcDNA3.1-Lhx8-FLAG), and the construct expressing bovine Figla-HA was generated by cloning the *Figla* ORF in frame with an HA tag sequence at the 3’-end into pcDNA3.1 (pcDNA3.1-Figla-HA). Both constructs were confirmed by sequencing.

HEK293 cells were maintained in Dulbecco modified Eagle medium supplemented with 10% (v/v) fetal bovine serum (Life technologies, Carlsbad, CA). Cells were seeded into 6-well plates at 5 x 10^5^ cells per well and transiently co-transfected with the expression constructs, pcDNA3.1-Lhx8-FLAG and pcDNA3.1-Figla-HA using X-tremeGENE 9 transfection reagent according to the manufacturer’s protocol (Roche Diagnostics). Cells were collected 24 h after transfection. Whole cell lysates were immunoprecipitated with anti-Flag antibody (Sigma-Aldrich, St. Louis, MO) using the Pierce Co-Immunoprecipitation kit according to the manufacturer’s instructions (Life Technologies). The co-immunoprecipitated proteins were analyzed by Western blotting with anti-HA antibody (Sigma-Aldrich). Protein detection was performed on an Odyssey system (Li-COR, Lincoln, NE).

## Results

### Cloning and analysis of bovine *Lhx8* and *Lhx8-v1* cDNA sequences

**Fig 1:**
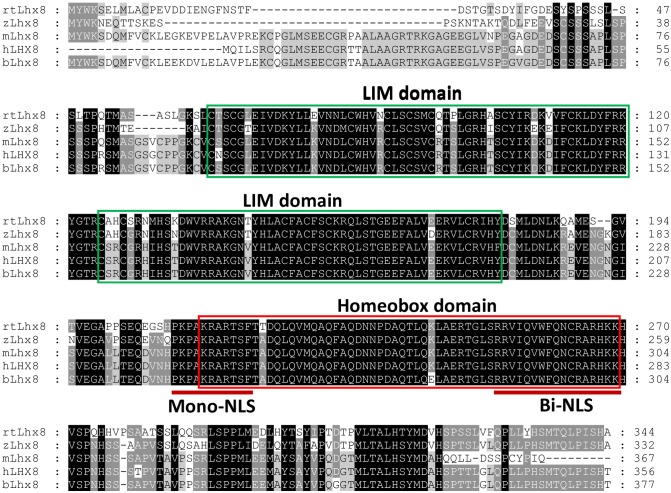
Multiple sequence alignment of Lhx8 proteins. Sequence alignment was performed using Clustal Omega (http://www.ebi.ac.uk/Tools/msa/clustalo/). The functional domains were determined by searching the Pfam database (http://pfam.xfam.org/search). The LIM and Homeobox domains are indicated by green and red boxes, respectively. mLhx8: mouse Lhx8 (NP_034843.2), hLhx8: human Lhx8 (NP_001001933.1), zLhx8: zebrafish Lhx8 (NP_001003980.1), rtLhx8: rainbow trout Lhx8 (unpublished data). bLhx8: bovine Lhx8 (KX898027).

**Fig 2:**
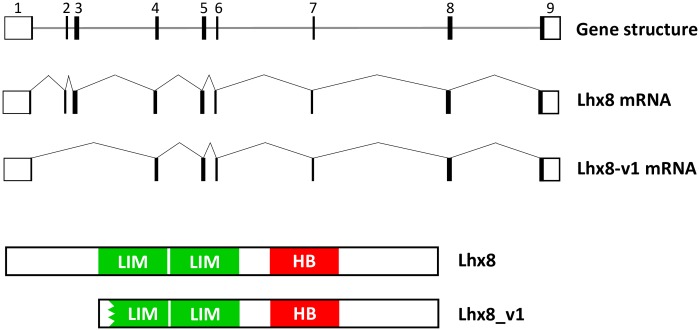
Structure of bovine *Lhx8* gene and its transcripts, *Lhx8* and *Lhx8_v1*. The bovine *Lhx8* gene contains 9 exons and spans about 28 kb. *Lhx8* encodes a protein of 377 amino acids. The splice variant, *Lhx8_v1* which results from alternative splicing of exon 2 and 3, codes for a protein of 293 amino acids. One of the LIM domains in the splice variant is incomplete due to deletion of 83 amino acids near the N terminus. LIM: LIM domain. HB: Homeobox domain.

The open reading frame (ORF) of bovine *Lhx8* gene was amplified from cDNA of a bovine fetal ovary using primers designed based on a predicted bovine *Lhx8* cDNA sequence (XM_010803408.1 X1) and a partial transcript obtained from bovine oocyte transcriptome sequencing. Additional 5’- and 3’-untranslated region (UTR) sequences overlapping the ORF were also PCR amplified. The assembled bovine *Lhx8* cDNA sequence is 1,807 bp in length, which has been deposited in the GenBank database with the accession number: KX898027. The predicted ORF of bovine *Lhx8 cDNA* encodes a protein of 377 amino acids. The deduced bovine Lhx8 protein contains two LIM domains and one homeobox domain, which are well conserved among multiple species including human, mouse, zebrafish and rainbow trout (Fig 1). In particular, bovine Lhx8 shares over 96% amino acid sequence identity with its human and mouse orthologs. BLAST search of the bovine reference genome sequence in the NCBI database using the bovine *Lhx8* cDNA identified the corresponding gene sequence, which is located on bovine chromosome 3. The bovine *Lhx8* gene contains 9 exons separated by 8 introns and spans about 28 kb (Fig 2). When analyzing the sequences of cloned ORF DNA fragments, a splice variant of *Lhx8* (*Lhx8_v1*) was identified, which results from alternative splicing of exon 2 and 3. *Lhx8_v1* encodes a protein of 293 amino acids, with one of the LIM domains being incomplete due to deletion of 83 amino acids near the N terminus (Fig 2). The cDNA sequence for *Lhx8_v1* has been deposited in the GenBank database with the accession number: KX898028.

### Expression of bovine *Lhx8* and *Lhx8-v1* mRNA in tissues and fetal ovaries

**Fig 3:**
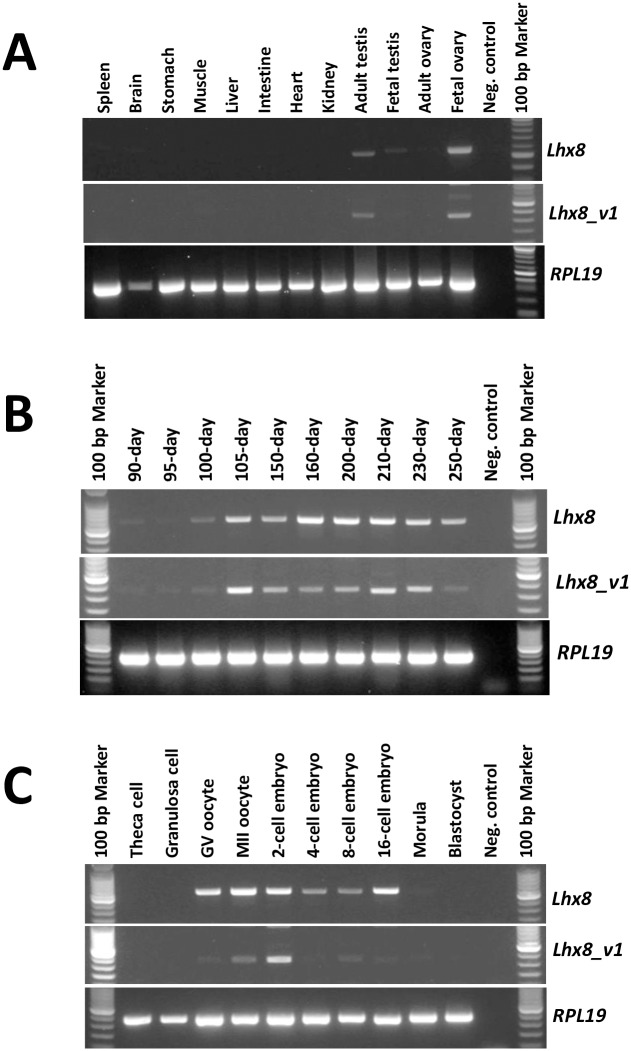
RT-PCR analysis of bovine *Lhx8* and *Lhx8-v1* mRNA expression. **A**: Expression of *Lhx8* and *Lhx8-v1* mRNA in bovine tissues. Tissues tested include spleen, stomach, brain, muscle, kidney, liver, heart, intestine, adult ovary, adult testis, fetal testis and fetal ovary. **B**: Expression of *Lhx8* and *Lhx8-v1* mRNA in bovine fetal ovaries from different gestation stages. Fetal ovaries from 90, 95, 100, 150, 160, 200, 210, 230 and 250 day fetuses were analyzed. The ages of fetuses were estimated based on crown-rump length. **C**: Expression of *Lhx8* and *Lhx8-v1* mRNA in oocytes and early embryos. Oocytes and embryos samples used in the analysis include GV- and MII-stage oocytes and 2-cell, 4-cell, 8-cell, 16-cell, and morula- and blastocyst-stage embryos. Bovine *RPL19* gene was used as a control for RNA quality.

Primers designed to specifically amplify either the *Lhx8* or the *Lhx8-v1* transcript were used in RT-PCR analysis of tissue distribution of *Lhx8* and *Lhx8-v1* transcripts. As shown in Fig 3A, both transcripts are predominantly expressed in fetal ovary and adult testis, although weak expression was also detected in fetal testis. By contrast, little or no expression was detected in any of the somatic tissues that were examined. Further RT-PCR analysis showed that *Lhx8* is not expressed in granulosa or theca cells (Fig 3C), suggesting that *Lhx8* expression in the fetal ovary is oocyte-specific. Analysis of expression of both transcripts in fetal ovaries of different developmental stages during gestation revealed that both transcripts can be detected in fetal ovaries as early as day 90 of gestation (Fig 3B) when primordial follicles emerge in cattle. The expression of both transcripts increases steadily in fetal ovaries during development, suggesting a role of this gene in the development of primary and secondary follicles which are formed around day 140 and 210 of gestation, respectively.

### Expression of bovine *Lhx8* and *Lhx8-v1* mRNA during oocyte maturation and early embryonic development

Temporal expression of bovine *Lhx8* and *Lhx8-v1* mRNA during oocyte maturation (GV- and MII-stage) and early embryonic development was examined by RT-PCR. *Lhx8* mRNA is abundant in GV and MII stage oocytes as well as in early stage (up to 16-cell) embryos but not detectable in morula and blastocyst stage embryos (Fig 3C). *Lhx8_v1* mRNA expression is detectable in oocytes and early embryo but not in morula and blastocyst stage embryos. The expression profiles of these two transcripts are different. Expression of *Lhx8_v1* appears to be lower, however, a relatively higher expression of *Lhx8_v1* was observed in 2-cell stage embryos (Fig 3C). These results suggest that bovine *Lhx8* is a maternal transcript abundantly present in oocytes and early embryos prior to zygotic genome activation.

### Subcellular localization of bovine Lhx8 protein

**Fig 4:**
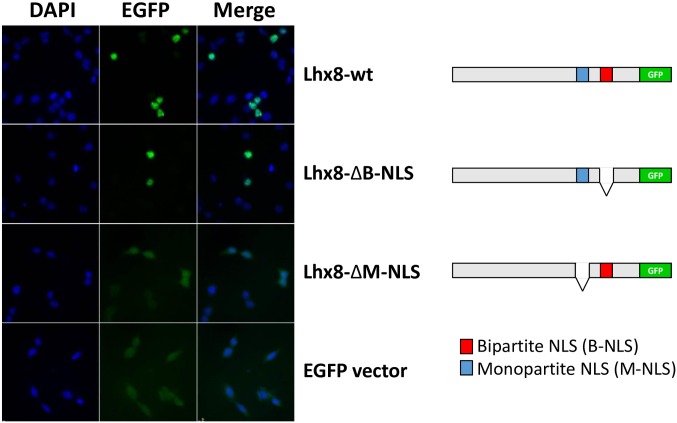
Cellular localization of bovine Lhx8 protein analyzed by a GFP reporter assay. HEK293 cells were transfected with GFP reporter constructs expressing either an EGFP-tagged wild type Lhx8 (Lhx8-wt) or Lhx8 mutants with either the monopartite NLS (Lhx8-ΔM-NLS) or the bipartite NLS deleted (Lhx8-ΔB-NLS). Empty pcDNA3-EGFP vector was used as a control. Nuclear DNA was stained with DAPI and cells were analyzed with a fluorescence microscope.

Using cNLS Mapper (http://nls-mapper.iab.keio.ac.jp/cgi-bin/NLS_Mapper_form.cgi) and pSORT II (http://psort.hgc.jp/form2.html) programs, bovine Lhx8 protein was predicted to possess two nuclear localization signals (NLS) indicating that it is a nuclear factor. One of the predicted NLSs is monopartite (position 243–253) and the other one is a bipartite NLS (position 287–303). To determine if bovine Lhx8 is mainly localized in the nucleus and which predicted NLS signal is required for its nuclear localization, we performed an EGFP reporter assay. HEK293 cells were transfected with plasmid constructs expressing either an EGFP-tagged wild-type Lhx8 protein or mutated Lhx8 proteins lacking either the monopartite NLS or the bipartite NLS. Fig 4 shows that the wild-type Lhx8 is exclusively localized to the nucleus of the transfected cells, while the mutant Lhx8 lacking the monopartite NLS is enriched in the cytoplasm. The mutant Lhx8 lacking the bipartite NLS still shows a nuclear localization. Thus Lhx8 is a nuclear protein and the predicted monopartite NLS is required for its nuclear localization.

### Evaluation of interactions of Lhx8 with Figla and Sohlh1

**Fig 5:**
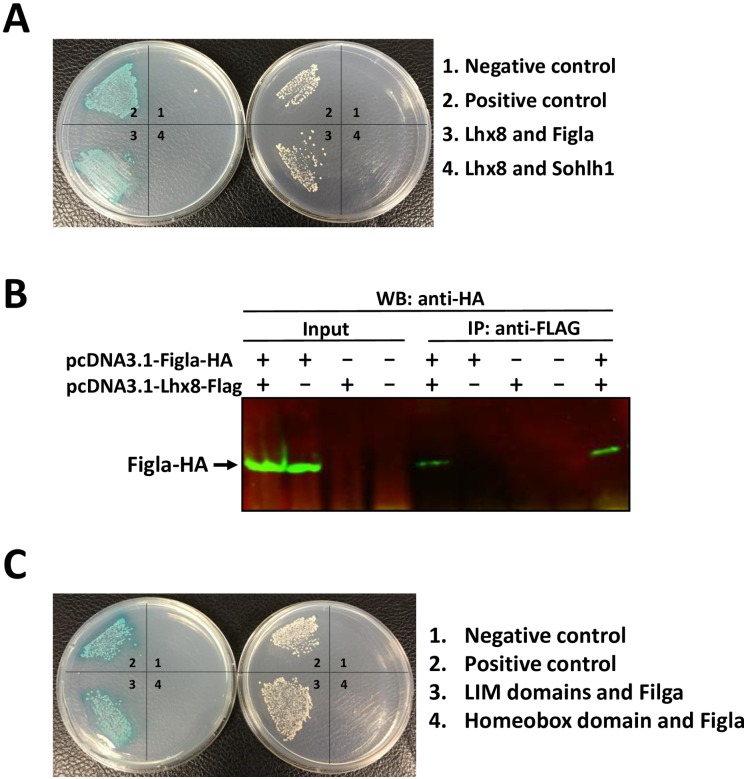
Interaction of Lhx8 with Figla. **A**: Yeast two-hybrid analysis of protein interactions between Lhx8 and Figla or Sohlh1. Right plate: Growth of yeast cells on DDO plate (medium lacking Leucine and tryptophan) showing successful co-transformations. Left plate: growth of yeast cells on selective QDO/X/A plate (Quadruple drop-out medium lacking leucine, tryptophan, adenine and histidine and containing X-gal) indicating protein-protein interactions. **B**: Co-IP analysis of interaction between Lhx8 and Figla. HEK293 cells were co-transfected with the expression constructs, pcDNA3.1-Lhx8-FLAG and pcDNA3.1-Figla-HA. Cell lysates were immunoprecipitated with anti-Flag antibody followed by Western blot analysis with anti-HA antibody. **C**: Direct yeast two-hybrid analysis showing LIM domains of Lhx8 are required for interaction with Figla. Right plate: Growth of yeast cells on DDO plate showing successful co-transformations. Left plate: growth of yeast cells on selective QDO/X/A plate indicating protein-protein interactions.

Both Figla and Sohlh1 are germ cell-specific transcription factors essential for early oogenesis. Both proteins contain a well conserved bHLH domain, which is likely to interact with LIM domain containing proteins. To determine if Lhx8 protein can interact with one or both of these two important nuclear proteins, we performed a direct yeast two-hybrid analysis. Yeast cells expressing GAL4-BD fused Lhx8 (bait) were mated with cells expressing GAL-AD tagged Figla or Sohlh1 (preys). Growth of yeast cells co-expressing GAL4 BD-Lhx8 and GAL4 AD-Figla fusion proteins was observed on selective QDO/X/A plate; however, no cell growth was observed on selective plate when yeast cells transformed with GAL4-BD-Lhx8 plasmid were mated with cells expressing GAL-AD-Sohlh1 fusion protein (Fig 5A). These results indicate that Figla but not Sohlh1 is a specific interacting partner of Lhx8.

We further performed Co-IP experiments to validate the interaction between Lhx8 and Figla. HEK293 cells, which do not express Lhx8 or Figla, were co-transfected with a construct expressing FLAG tagged Lhx8 and a construct expressing HA tagged Figla. Two days after transfection, the cells were lysed under mild condition and immunoprecipitated with an anti-FLAG antibody. The co-immunoprecipitated proteins were analyzed by Western blotting with an anti-HA antibody. As shown in Fig 5B, Figla-HA was co-immunoprecipitated with Lhx8-FLAG only when both tagged proteins were expressed, confirming the formation of protein complex between Lhx8 and Figla.

### Lhx8: Figla interaction is dependent on the LIM domains of Lhx8

Previous studies have shown that most LIM domains are responsible for protein-protein interactions, and rarely do homeobox domains interact with other proteins. To determine which functional domains of Lhx8 is responsible for Lhx8: Figla interaction, we performed a direct yeast two-hybrid analysis using constructs expressing GAL4 BD-Lhx8 fusion proteins with either the LIM domains or the homeobox domain deleted (GAL4 BD-ΔLIM and GAL4 BD-ΔHB, respectively). The mutant GAL4 BD-Lhx8 fusion proteins were evaluated for their ability to interact *in vivo* with the GAL4 AD-Figla fusion protein. Growth of yeast cells expressing GAL4 BD-ΔHB and GAL4 AD-Figla was observed on selective QDO/X/A plate. By contrast, no growth was detected for yeast cells expressing GAL4 BD-ΔLIM and GAL4 AD-Figla, indicating that the LIM domains, but not the homeobox domain, are required for Lhx8: Figla interaction (Fig 5C).

## Discussion

In the present study, we show that expression of bovine *Lhx8* mRNA is restricted to gonadal tissues, and is temporally regulated during early embryonic development. We also show that Lhx8 and Figla, two transcription factors that are crucial for oogenesis, interact directly. This is the first time that a direct protein-protein interaction between two germ cell-specific nuclear transcription factors is discovered.

Oogenesis is a tightly controlled process that require spatial and temporal regulation of genes involved in several regulatory pathways. Transcription factors that contain LIM domain and bHLH domain play critical roles during the process. These transcription factors selectively regulate target genes by context-dependent protein-protein interactions [20]. In this study, we show that Lhx8 interacts with Figla, which is another germ cell-specific transcription factor essential for oogenesis. The specificity of the interaction between the two proteins implies a functional relationship between the two proteins and represents an example of protein-protein interactions mediated by the LIM and bHLH motifs. The function of Lhx8:Figla interaction may include recruitment of transcriptional coactivators or corepressors leading to transcriptional activation or repression, thereby maintaining a stringent control over progressive growth and differentiation of the oocyte during early development. Our results also show that Lhx8: Figla interaction is dependent on the LIM domains of Lhx8. This is consistent with previous findings showing that the LIM domains of LIM homeobox proteins are the components that function in protein-protein interactions which specify homeobox domain binding to DNA and regulate the transcriptional activity subsequently [9, 15, 21, 22].

Maternal transcripts and proteins that are accumulated in the oocyte during oogenesis play crucial roles during initial development of early embryos, before embryonic genome activation [23]. Some maternal transcripts show oocyte-specific expression and they are known as maternal effect genes, which are essential for the early cleavage of embryos [24–26]. In cattle, a number of oocyte-specific maternal effect genes have been identified, which include JY-1 [27], Kpna7 [28] and Nobox [29]. These maternal effect genes share a very similar expression pattern during early embryonic development. The transcripts of these genes are highly abundant in oocytes and early stage embryos, but barely detectable or undetectable in morula and blastocyst stage embryos. The expression pattern of bovine *Lhx8* mRNA during early embryogenesis is very similar to these known bovine maternal-effect genes, suggesting that bovine Lhx8 is not only an essential factor for oocyte/follicular development, but also an important regulator for the development of early embryos.

Many nuclear proteins contain typical NLSs that are recognized by importin α/importin β heterodimers for nuclear transport [30]. There are two types of well characterized NLSs used by the importin α/importin β transport system. A monopartite NLS consists of a single stretch of basic amino acids, and a bipartite NLS contains two stretches of basic amino acids separated by a spacer of 10 to 37 amino acids [31]. Bovine Lhx8 was predicted to contain both types of NLSs. However, our results show that the monopartite NLS plays the major role for nuclear translocation of this protein. Some Lhx8 protein can still translocate into the nucleus without the monopartite NLS, suggesting that the monopartite NLS is required but not necessary. Thus, other than the well characterized classical nuclear translocation mechanism, there should be other pathways that facilitate this important germ cell-specific transcription factor to go into the nucleus. Further studies of the mechanisms governing its nuclear translocation are needed.

In summary, we have characterized the bovine *Lhx8* gene and analyzed its expression during oocyte maturation and early embryogenesis. We have also demonstrated that Lhx8 interacts with Figla, indicating that these two germ cell-specific transcription factors may function in a cooperative fashion during early oocyte development.
